# *In Vitro* Activities of Omadacycline and Comparators against Anaerobic Bacteria

**DOI:** 10.1128/AAC.00047-18

**Published:** 2018-03-27

**Authors:** Laure Stapert, Cindy Wolfe, Dean Shinabarger, Andrea Marra, Chris Pillar

**Affiliations:** a Micromyx, Inc., Kalamazoo, Michigan, USA

**Keywords:** anaerobes, omadacycline

## Abstract

Omadacycline (OMC), a broad-spectrum aminomethylcycline, has shown clinical efficacy in anaerobic acute bacterial skin and skin structure infections (ABSSSI) and in animal models of intra-abdominal anaerobic infections. Here, the *in vitro* activity of OMC against clinically relevant anaerobes was similar to that of tigecycline, with MIC_90_ values of 1 to 8 μg/ml against Bacteroides spp., 0.5 μg/ml against Clostridium difficile, Prevotella spp., and Porphyromonas asaccharolytica, 1 μg/ml against Peptostreptococcus spp., and 16 μg/ml against Clostridium perfringens.

## TEXT

In nature, anaerobic bacteria are ubiquitous organisms, of which a diverse array exists as part of the normal human microflora associated with mucous membranes (1, 2). A variety of anaerobic infections can occur, typically due to disruption of this commensal relationship with the host, and involve a comparatively less diverse group of organisms upon breach of a mucous membrane barrier at or near the site of infection. These infections are frequently polymicrobial and usually result in abscess formation (1, 2). Anaerobic infections are most often treated with β-lactams plus β-lactamase inhibitors, metronidazole (MTZ), clindamycin (CLI), carbapenems, tigecycline, and/or cefoxitin (1, 2). A novel aminomethylcycline, omadacycline (OMC), has activity against the two most common tetracycline resistance mechanisms and is currently undergoing clinical evaluation by Paratek Pharmaceuticals (Boston, MA) for the treatment of ABSSSI and community-acquired bacterial pneumonia (3). In ABSSSI trials and in animal models of anaerobic infection (e.g., intra-abdominal infection), OMC has demonstrated efficacy against anaerobic infections (4, 5).

(Findings from this study were presented at the 27th European Congress of Clinical Microbiology and Infectious Diseases (ECCMID), held in Vienna, Austria, from April 22 to 25, 2017.)

The activities of OMC and comparators were evaluated against the following anaerobic organisms from the Micromyx repository (*n* = 186; Tables 1 and 2): Bacteroides fragilis, Bacteroides thetaiotaomicron, Bacteroides vulgatus, Bacteroides ovatus, Clostridium difficile, Clostridium perfringens, Peptostreptococcus spp., Porphyromonas asaccharolytica, and Prevotella spp. The test organisms consisted of randomly selected, nonconsecutive, nonduplicate human clinical isolates collected from 2006 to 2016 within the United States; most of the isolates were from abscesses, wounds, or infections of the gallbladder, blood, or abdomen. C. difficile isolates were isolated from stool samples. Nine of the evaluated P. asaccharolytica isolates were veterinary in origin, collected in 2007 in Japan. OMC powder was provided by Paratek and was stored at −80°C. Comparator drugs included tigecycline (TGC), meropenem (MEM), moxifloxacin (MXF), CLI, MTZ, and piperacillin-tazobactam (TZP). Stock solutions of these reference compounds were prepared on each day of the assay using solvents recommended by the Clinical and Laboratory Standards Institute (CLSI) (6, 7). Concentration ranges used during testing spanned relevant quality control ranges and breakpoints established for each test compound against anaerobes (6, 7). Tazobactam was tested at a fixed concentration of 4 μg/ml, in combination with piperacillin.

**TABLE 1:**
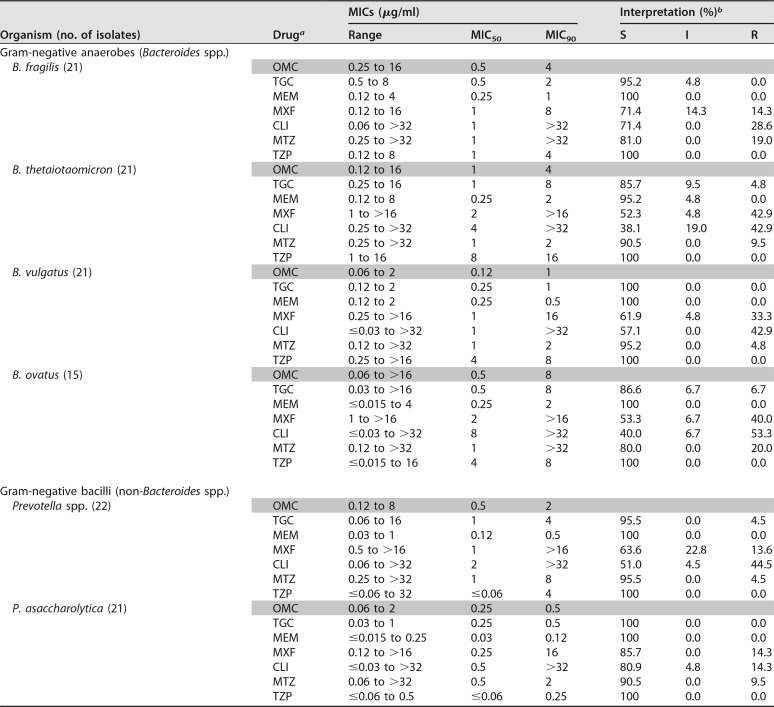
Summary of the *in vitro* activity of OMC and comparators against anaerobes

a OMC, omadacycline; TGC, tigecycline; MEM, meropenem; MXF, moxifloxacin; CLI, clindamycin; MTZ, metronidazole; TZP, piperacillin-tazobactam (tazobactam was tested at a constant concentration of 4 μg/ml; the piperacillin MICs are shown).

b MIC values were interpreted based on CLSI breakpoints (6) except for those of tigecycline, which were interpreted based on FDA prescribing information for Tygacil (7). S, susceptible; I, intermediate; R, resistant.

**TABLE 2:**
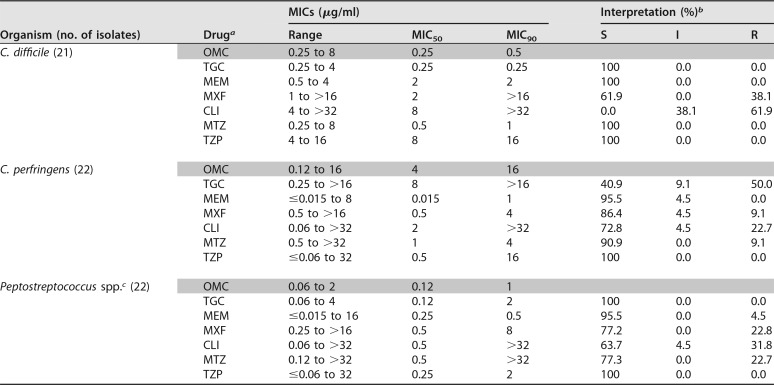
Activity of OMC and comparators against Gram-positive anaerobes

a For TZP, tazobactam was tested at a constant concentration of 4 μg/ml; the piperacillin MICs are shown.

b MIC values were interpreted based on CLSI breakpoints (6), except for those of tigecycline, which were interpreted based on FDA prescribing information for Tygacil (7).

c Peptostreptococcus spp. included 11 Peptostreptococcus micros and 11 Peptostreptococcus anaerobius isolates.

For Bacteroides spp. only, MIC determinations were made by broth microdilution; all other organisms were evaluated by agar dilution and all testing was performed in accordance with CLSI guideline M11-A8 (6) and CLSI supplement M100-S26 (7), using freshly prepared Brucella broth and agar. Where noted, MIC values were interpreted as susceptible (S), intermediate (I), or resistant (R), in accordance with CLSI supplement M100-S26 (7), with the exception of TGC, where FDA interpretive criteria were used (8). Relevant quality control (QC) isolates from the American Type Culture Collection (ATCC; Manassas, VA) (B. fragilis ATCC 25285, B. thetaiotaomicron ATCC 29741, and C. difficile ATCC 700057) were included during testing. MIC values for QC isolates were within established quality control ranges for all drugs.

As shown in Table 1, OMC demonstrated potent activity relative to that of comparator agents against Bacteroides spp., including B. fragilis, B. thetaiotaomicron, B. vulgatus, and B. ovatus; MIC_50/90_ values for OMC against these organisms were 0.5/4, 1/4, 0.12/1, and 0.5/8 μg/ml, respectively. OMC was also active against Prevotella spp. and P. asaccharolytica, with MIC_50/90_ values of 0.5/2 and 0.25/0.5 μg/ml, respectively (Table 1).

Against the Gram-positive anaerobes C. difficile and Peptostreptococcus spp., OMC also demonstrated potent activity, with MIC_50/90_ values of 0.25/0.5 and 0.12/1 μg/ml, respectively (Table 2). However, against C. perfringens OMC was less active, with MIC_50/90_ values of 4/16 μg/ml (Table 2).

Overall, the evaluated isolates were found to be susceptible to TZP in this study, and most were susceptible to MEM and TGC (with the exception of C. perfringens to TGC, 40.9% S) (Tables 1 and 2). As expected, MTZ also showed good activity, with >90% S across species, except for B. fragilis (81% S), B. ovatus (80% S) and Peptostreptococcus spp. (77.3% S) (Tables 1 and 2). As expected, CLI and MXF had fairly poor activity in this study, with susceptibilities in the range of 38.1 to 70% for the Bacteroides spp. and 0 to 86.4% for the Clostridium spp. (Tables 1 and 2).

In conclusion, OMC had potent activity *in vitro* against Gram-negative and Gram-positive anaerobes commonly isolated from human infections. The activity of OMC against anaerobes was similar to that reported previously (3) and also parallels that observed with TGC, an agent indicated for the treatment of anaerobes in skin and intra-abdominal infections (8), by both MIC_50/90_ and MIC distribution, with values identical or within 2-fold (Tables 1 and 2). The *in vitro* activity of OMC against anaerobic pathogens, along with the *in vivo* efficacy against anaerobes in animal models of anaerobic infection and in human skin infections, highlights the potential of OMC for the treatment of human anaerobic infections.
